# Factors determining the patients’ care intensity for surgeons and surgical nurses: a conjoint analysis

**DOI:** 10.1186/s12913-015-1052-4

**Published:** 2015-09-18

**Authors:** Catharina J. van Oostveen, Hester Vermeulen, Els J. M. Nieveen van Dijkum, Dirk J. Gouma, Dirk T. Ubbink

**Affiliations:** Department of Surgery, Academic Medical Center, P.O box 22700, 1100 DE Amsterdam, The Netherlands; Amsterdam School of Health Professions, University of Amsterdam, P.O box 22700, 1100 DE Amsterdam, The Netherlands

**Keywords:** Conjoint analysis, Physician intensity, Nurse intensity, Surgery, Surgical wards, Patient care intensity, Hospitalization, Vignettes, Clinical scenarios, Hospital resources, Doctors, Nurses, Surgeons, Physicians, Questionnaire, Demand for care

## Abstract

**Background:**

Surgeons and nurses sometimes perceive a high workload on the surgical wards, which may influence admission decisions and staffing policy. This study aimed to explore the relative contribution of various patient and care characteristics to the perceived patients’ care intensity and whether differences exist in the perception of surgeons and nurses.

**Methods:**

We invited surgeons and surgical nurses in the Netherlands for a conjoint analysis study through internet and e-mail invitations. They rated 20 virtual clinical scenarios regarding patient care intensity on a 10-point Likert scale. The scenarios described patients with 5 different surgical conditions: cholelithiasis, a colon tumor, a pancreas tumor, critical leg ischemia, and an unstable vertebral fracture. Each scenario presented a mix of 13 different attributes, referring to the patients’ condition, physical symptoms, and admission and discharge circumstances.

**Results:**

A total of 82 surgeons and 146 surgical nurses completed the questionnaire, resulting in 4560 rated scenarios, 912 per condition. For surgeons, 6 out of the 13 attributes contributed significantly to care intensity: age, polypharmacy, medical diagnosis, complication level, ICU-stay and ASA-classification, but not multidisciplinary care. For nurses, the same six attributes contributed significantly, but also BMI, nutrition status, admission type, patient dependency, anxiety or delirium during hospitalization, and discharge type. Both professionals ranked ‘complication level’ as having the highest impact.

**Discussion:**

The differences between surgeons and nurses on attributes contributing to care intensity may be explained by differences in professional roles and daily work activities. Surgeons have a medical background, including technical aspects of their work and primary focus on patient curation. However, nurses are focused on direct patient care, i.e., checking vital functions, stimulating self-care and providing woundcare.

**Conclusions:**

Surgeons and nurses differ in their perception of patients’ care intensity. Appreciation of each other’s differing interpretations might improve collaboration between doctors and nurses and may help managers to match hospital resources and personnel.

**Electronic supplementary material:**

The online version of this article (doi:10.1186/s12913-015-1052-4) contains supplementary material, which is available to authorized users.

## Background

Western European and North American hospitals are forced to improve efficiency while using limited available resources. This increases pressure on the overall quality of patient care, but also on the caregivers involved. Physicians and nurses form the main body of caregivers in the hospital setting. Hence, optimum staffing of physicians and nurses is crucial for patient safety, staff working conditions, retention and hospitalization costs [1, 2]. Patients receiving insufficient care due to high workload because of insufficient staffing are at risk for higher morbidity and mortality rates [3–5]. A high workload also has a significant impact on job satisfaction [6].

Nowadays, financial arguments mainly determine the staffing of physicians and nurses in European hospitals [7, 8]. Subsequently, many physicians and nurses perceive a high workload [9–11] and inefficient teamwork, as their perceived workload seems different and not transparent [11]. However, it is hard to substantiate this with objective measures that are intelligible for both disciplines as well as managers.

The caregivers’ workload is generally determined by the demand for care, personnel characteristics and orga-nizational characteristics [12]. Several studies searched for predictors of the demand for medical and nursing care. In a recent systematic review no accurate models were found, although some separate predictors appeared useful [13]. However, these predictors explained the demand for care in terms of required resources and hospitalization costs rather than physician or nurse staffing or workload [14]. For nurses, attempts have been made to match the patients’ demand for nursing care with nurse staffing supplies, for instance by measuring patient-related workload. This workload is the result of the demand for nursing care, and is defined here as (nursing) care intensity [15]. Little is known about the care intensity physicians perceive and which factors they believe influence care intensity. It is also unclear whether clinicians, in particular physicians and nurses, perceive this care intensity in the same way, as they are involved in other aspects of hospitalized patient care.

Workload is of particular importance for physicians and nurses working on surgical wards, because more than 50 % of adverse events are related to surgical procedures [16]. Therefore the risks of direct harm and high hospitalization costs are substantial for surgical wards. Hence, we investigated the perceptions of surgeons and nurses working in clinical settings regarding determining factors of care intensity. For this purpose we used a conjoint analysis (CA), presenting the clinicians 20 scenarios of hospitalized patients with five surgical conditions. In doing so, we attempted to detect the relative contribution of various patient and care characteristics to the perceived patients’ care intensity and possible differences in appreciation between surgeons and nurses.

## Methods

The conduct and description of this study was done according to the International Society for Pharmacoeconomics and Outcomes Research (ISPOR) checklist for conjoint analysis (CA) applications in healthcare research [17]. Our local medical ethics review board (Academic Medical Center, Amsterdam, The Netherlands) approved the study but waived the need for ethical approval as the study had no effect on the participants’ wellbeing. Yet, all participants received an explanation about the study and gave consent by participating in the study.

### Design

We performed a CA study in which participants were to appraise the caring intensity of fictional hospitalized patient scenarios (“vignettes”). These scenarios share the same set of so-called attributes (e.g., age), but the levels (e.g., below or above 65 years) of each attribute vary across the different scenarios.

Originally, CA is a method of eliciting consumer preferences in marketing research, and allows estimation of the relative importance of different characteristics (attributes) for the valuation of rate descriptions of a good. This method has also been applied successfully in the realm of healthcare [18].

Each scenario described a patient admitted to a surgical ward and characterized by thirteen attributes. These attributes represent various patient and care characteristics, e.g., age, polypharmacy, complication level, and American Society of Anesthesiologists classification (ASA class) (Table 1). Possible relevant attributes were derived from a systematic review and a pilot study on the use of hospital care services of surgical patients [13, 14].

**Table 1:** Attributes used in the scenario’s

Attribute	Levels	N
Age	0 = <65	10
	1 = >65	10
BMI	0 = <30	14
	1 = >30	6
Nutrition status^a^	0 = no	12
	1 = yes	8
Polypharmacy	0 = <5	10
	1 = >5	10
Medical diagnosis	Cholelithiasis	4
	Colon tumor	4
	Pancreas tumor	4
	Critical leg ischemia	4
	Unstable vertebral fracture	4
Admission type	0 = planned	13
	1 = emergency	7
ASA-classification	I	5
	II	6
	III	9
Patient dependency	0 = independent	6
	1 = partially dependent	11
	2 = totally dependent	3
Complication level	0 = no complication	5
	1 = any deviation from the normal postoperative course	6
	2 = requiring pharmacological treatment	5
	3 = requiring surgical, endoscopic or radiological intervention	4
Anxiety or delirium	0 = no	14
	1 = yes	6
ICU stay	0 = no	15
	1 = yes	5
Multidisciplinary treatment^b^	0 = no	8
	1 = yes	12
Discharge type	0 = home	12
	1 = other	8

*N* number of times used in vignettes

^a^>10 % loss bodyweight within 6 months

^b^>1 discipline involved in treatment

All attributes were divided into an appropriate number of levels, e.g., ASA classes I, II, and III. However, the number of levels within each attribute was kept to a minimum in order to limit the number of scenarios required to present a representative range of different scenarios.

Subsequently, a small group of 6 surgeons and 8 surgical nurses (with different specialty and experience) were invited to a single Delphi round [19], in order to generate a set of apparently influencing characteristics. The contents of the final scenarios were generated by the orthogonal design in SPSS (Statistical Package for the Social Sciences v. 20; IBM SPSS Inc., Armonk, NY, USA).

### Setting and participants

Surgeons and surgical nurses from university centers, teaching hospitals and community hospitals in the Netherlands were invited to participate in the questionnaire. Surgeons, including residents, belonging to the regional surgical resident teaching program educational area of the Academic Medical Center were invited via e-mail. A total of two reminders were sent to reach a response rate of 60 %.

Surgical nurses were approached by advertising in three Dutch nursing journals (both paper and web pages), a call on a LinkedIn forum, Twitter, Facebook, and the science webpage of one of the teaching hospitals.

### Conjoint analysis questionnaire

The researchers eventually selected 20 plausible scenario’s for data collection. This is an ample number as compared to current literature [20, 21]. An example of the composition of the scenarios is given in Table 2, and a full example of a scenario is given in Additional file 1. A pilot study was conducted among four surgeons and two nurses to test whether any vital information in the 20 scenarios was missing. This was corrected if necessary.

**Table 2:** Example of the composition of five scenarios and the levels of each attribute used

	Attributes												
Vignette number	Diagnosis	Age	Poly- pharmacy	Complication level	ASA class	BMI	ICU-stay	Nutrition status	Admission type	Discharge type	Patient dependency	Anxious or delirious	Multi-disciplinary treatment
1	Cholelithiasis	1	1	3	III	0	1	0	0	0	1	0	1
5	Critical leg ischemia	1	1	2	III	1	0	0	0	0	1	0	1
9	Colon tumor	0	0	3	II	0	1	0	0	1	1	0	1
13	Pancreas tumor	1	0	1	II	0	0	1	0	0	2	1	0
17	Unstable vertebral #	0	0	2	II	0	0	0	1	1	1	1	1

The contents of the levels corresponding with numbers 1–3 are shown in Table 1

By means of a digital questionnaire (www.Surveymonkey.com), the surgeons and surgical nurses were asked to score the care intensity of the 20 scenarios on a 10-point Likert scale, ranging from 0 (“very low intensity”) to 10 (“very high intensity”). In addition surgeons and nurses were asked to state their top-5 of attributes contributing most to care intensity.

### Statistical analysis

Continuous variables regarding the respondents’ characteristics and scenario scores were expressed as means and standard deviations (SD). The relative importance of each of the attributes as to the perceived care intensity was determined by means of a fixed effect linear multi-level analysis to account for the multilevel structure of the scenarios (level 1) as rated by surgeons and nurses (level 2).

The effect size of each attribute was expressed as a β-coefficient with its 95 % confidence interval (CI). β-coefficients above or below 0 (the reference value) indicate the attribute contributes to a higher or lower care intensity score, respectively. The attribute top-five for surgeons and nurses was assessed based on their mean rating scores per attribute. *P*-values <0.05 were considered significant.

## Results

A total of 82 surgeons and 146 surgical nurses completed the questionnaire, resulting in 4560 rated scenarios, 912 per condition. Characteristics of the responding surgeons and nurses are summarized in Table 3. The majority of the surgeons was male, with a mean age of 46.4 (SD 9.7), while the majority of the nurses was female; mean age 33.2 (SD 12.0). Most respondents worked in a tertiary referral hospital and were employed in trauma or gastro-intestinal surgery. No significant associations between surgeons’ or nurses’ characteristics (i.e., age, gender, surgical specialty of employment, years of experience, or hospital type) and care intensity judgments were observed.

**Table 3:** Characteristics of participants

	*N* (%)	*N* (%)
	Nurses (*N* = 146)	Surgeons (*N* = 82)
Gender (male)	12 (8.2)	65 (72.3)
Age	33.3 (range 20–62)	46.4 (range 28–66)
Experience (yrs)	11.4 (range <1–40)	16.8 (range 1–40)
Hospital		
Academic	21 (14.4)	18 (22.0)
Tertiary/educational	69 (47.3)	47 (57.3)
General	56 (38.4)	17 (20.7)
Specialty		
Vascular	30 (20.5)	14 (17.1)
Trauma	43 (29.5)	15 (18.3)
Gastro-intestinal	18 (12.9)	33 (40.2)
General	13 (8.9)	20 (24.4)
Orthopedic	10 (6.8)	0 (0)
Plastic	3 (2.1)	0 (0)
Urology	5 (3.4)	0 (0)
Other	24 (16.4)	0 (0)

### Attribute weights

The overall mean care intensity scores for the 20 scenarios were 6.21 (SD 2.08) among surgeons and 5.76 (SD 2.26) among nurses, which did not differ significantly.

**Fig. 1:**
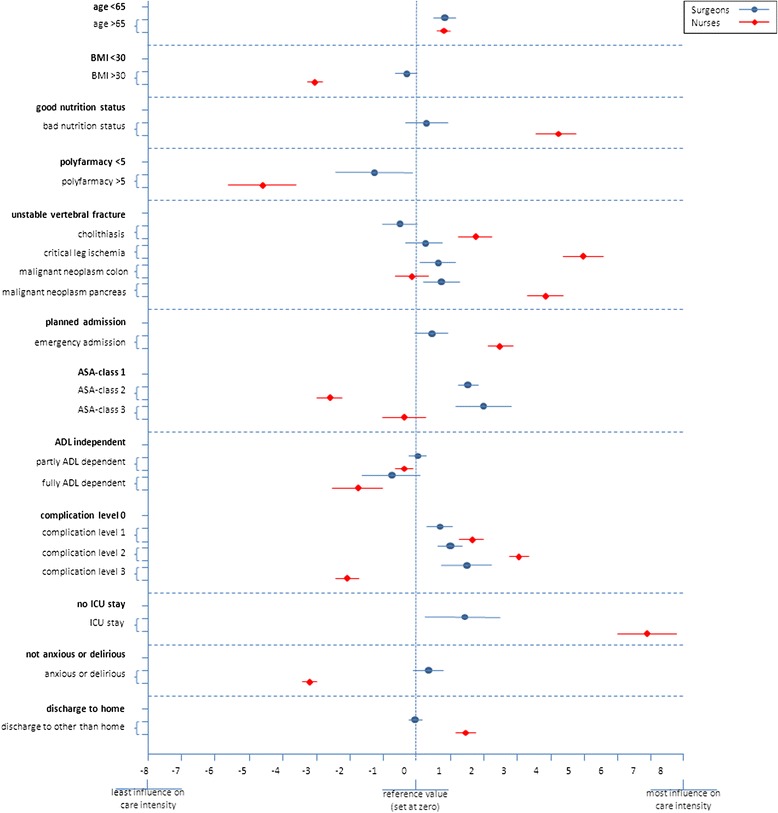
Contribution to care intensity of each of the attributes as perceived by surgeons and nurses (expressed as beta-coefficients and 95 % CI)

Figure 1 shows the contribution to care intensity of the significant attributes as perceived by surgeons and nurses.

#### Surgeons

According to the surgeons, 6 out of the thirteen attributes significantly influenced care intensity; age, polypharmacy, medical diagnosis, ASA-classification, complication level, and Intensive Care Unit (ICU) stay.

Surgeons assigned significantly more caring intensity to ASA classes II and III (β1.51; *p* < 0.001, and β2.12; *p* < 0.001, respectively), patients suffering from more severe complications (β1.58; *p* < 0.001), and ICU-stay (β1.36; *p* = 0.016). Furthermore, patients using less than 5 medications (β-1.30; *p* = 0.037), patients with medical diagnoses as colon or pancreatic cancer (β0.63; *p* = 0.017 and β0.72; *p* = 0.013), and those above 65 years of age (β0.83; *p* < 0.001) were also perceived as more care intensive.

#### Nurses

All except one attribute significantly influenced the judgments of the scenarios for nurses. Again, the attribute ‘multi-disciplinary treatment’ was redundant.

Nurses perceived ICU-stay as most care-intensive (β6.92; *p* < 0.001), followed by patients diagnosed with critical leg ischemia (β5.06; *p* < 0.001), a bad nutrition status (β4.23; *p* < 0.001) pancreas cancer (β3.93; *p* < 0.001), a complication at level 2 (β3.11; *p* < 0.001), and an emergency admission (β2.53; *p* < 0.001). Being 65 or older, having a diagnosed cholelithiasis, a complication level 1 (β1.78; *p* < 0.001) and sending home after discharge (β1.39; *p* < 0.001) contributed slightly but significantly more to care intensity. Surprisingly, patients classified as ASA II (but not ASA III) were considered less care-intensive than patients classified as ASA I. Furthermore, partly or totally dependent patients were perceived as less care-intensive than independent patients (β-0.44; *p* = 0.004, and β-1.79; *p* < 0.001). Patients with complications were assigned higher care intensity. However, patients with the most severe complications (apart from mortality), for instance patients with polyneuropathy, were considered to be less care-intensive (β-2.01; *p* < 0.001) as well as anxious and delirious patients (β3.33; *p* < 0.001).

### Priority scores

Fifty-nine (72 %) surgeons and ninety-four (64 %) nurses gave their ranking. Both surgeons and nurses indicated that the occurrence of complications was most influential for care intensity (Table 4). Three out of the top-five of attributes were the same among surgeons and nurses, i.e., medical diagnosis, complication level and ICU-stay. However, the mean ratings differed: the surgeons’ mean score for medical diagnosis in their top-five was 3.73, as compared to 5.85 for nurses.

**Table 4:** Top 5 and mean priority scores of attributes as perceived by surgeons and nurses

Top 5 (surgeons)	Priority scores
1. Complication level	2.05
2. Medical diagnose	3.73
3. ICU stay	4.25
4. ASA-class	5.61
5. Age	6.00
Top 5 (nurses)	
1. Complication level	2.94
2. Anxious or delirious	3.46
3. Medical diagnosis	5.85
4. Patient dependency	5.90
5. ICU stay	5.97

## Discussion

This study shows that 13 attributes contribute to a patient’s care intensity according to both surgeons and nurses; age, polypharmacy, medical diagnosis, complication level, ICU-stay and ASA-classification. In general, nurses assigned more weight to these attributes than surgeons. In addition, nurses also considered BMI, nutrition status, admission type, patients’ physical dependency, anxiety or delirium during hospitalization and discharge type as important factors influencing caring intensity.

The differences between surgeons and nurses on which attributes contribute to care intensity may be explained by differences in professional roles and daily work activities. Surgeons have a medical background, including technical aspects of their work and primary focus on patient curation and the direct results of the surgical procedure, which involves (multi-specialist) discussions on high risk patients and planning diagnostic or surgical interventions [22]. Nurses on the other hand are more focused on direct patient care, i.e., checking their vital functions, stimulating patients towards self-care, providing wound care and the related documentation [23]. Tasks which are substantial for patients diagnosed with critical leg ischemia. Furthermore, estimating care intensity can be difficult as its concept tends to be confounded with the concept’care complexity’. As nurses assigned less care intensity to patients classified as ASA II, patients with lasting damage due to a complication (level 3), and patients who were partly or totally dependent or had a delirium, the suspicion rises that nurses appreciated care complexity rather than care intensity, e.g., they only scored highly what they thought was beyond their routine and complex work to perform. Surgeons, in contrast, seemed to focus more on the workload and consequences they anticipate with increasing disease severity (higher ASA-class, more complex surgical interventions, more postoperative complications, more need for intensive care). Overall, nurses assigned more extreme weights than surgeons. This raised the suspicion that nurses appreciated care complexity instead of care intensity.

To our knowledge, this is the first study on the interpretation of the patients’ care intensity by surgeons and on the comparison between surgeons and nurses as to the care intensity they perceive. Awareness of the factors contributing to caring intensity is of major relevance to hospital managers who aim at optimizing the care processes on clinical wards. First, because this information helps managers to align the surgeons’ and nurses’ organizational processes and even tailor their personnel and resources on the wards. For instance, the finding that more attributes play a role for nurses than for surgeons regarding the patients’ care intensity should be an important new criterion for planners of patient admissions, since this apparently depends on several factors that may be different from, and on top of, those perceived by surgeons.

Second, knowing these differences may help surgeons and nurses to understand each other’s care intensity criteria, and better synchronize their patient care. Understanding, appreciating and respecting each other’s work has a positive impact on patient safety and provides learning possibilities for caregivers as well as improving working conditions [24, 25].

Some limitations of our study merit discussion. Not all characteristics that influence the patients’ caring intensity could be included in the scenarios. For instance, comorbidities or an Early Warning Score (EWS) to account for physical deterioration could not be taken into account. However, as we conducted a single Delphi round before creating the scenarios, these factors were not considered as influential. Moreover, these attributes are highly correlated with polypharmacy and ICU-stay, which are more suitable attributes because they contain fewer levels. Another suitable attribute would have been the Charlson comorbidity index as a weighted measure for patient comorbidity [26]. However, this measure is, besides in the Hospital Standard Mortality Rate (HSMR), not commonly used for registration in the surgical departments in the Netherlands, and was therefore not included. Furthermore, only the main effects of the attributes could be measured. Possible interactions between main effects are unknown, e.g., between age and ASA-classification, or between age and polypharmacy. Finally, the caregivers’ workload is not only determined by demand for care, but also by personnel and organizational factors [12]. The latter two factors were not included in this study, because too few personnel and organizational factors could be collected to adequately address this issue.

## Conclusion

According to surgeons and nurses, six patient-related factors influence the care intensity of hospitalized surgical patients, of which ‘complication level’ ranked highest. Nurses also considered another six factors as important, possibly due to the nature of their daily work activities and the way they interpreted care intensity. It is worthwhile to explore the (differences in) perceived care intensity by physicians and nurses in different populations and healthcare organizations. Therefore, the results need to be further explored, but awareness of these factors may help managers optimize the work processes on nursing wards, in terms of staff planning and aligning the activities of surgeons and nurses. Furthermore, surgeons and nurses may better appreciate each other’s care intensity. Future research is needed to explore whether an objective measure for care intensity can foster this and may positively affect patient safety on clinical wards.

## Additional file

Additional file 1:
**Clinical scenario.** (PDF 118 kb)
