# Supplementary figures and images for: Factors determining the patients’ care intensity for surgeons and surgical nurses: a conjoint analysis
Source: BMC Health Serv Res. 2015 Sep 18;15:395. doi: 10.1186/s12913-015-1052-4 (PMC4575441; doi:10.1186/s12913-015-1052-4)

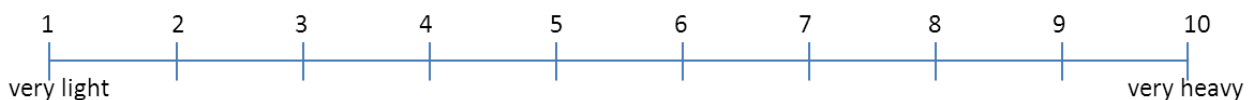

Supplement: Additional file 1: — Clinical scenario. (PDF 118 kb) [file 12913_2015_1052_MOESM1_ESM.pdf]
